# The Impact of Optimized Daylight and Views on the Sleep Duration and Cognitive Performance of Office Workers

**DOI:** 10.3390/ijerph17093219

**Published:** 2020-05-06

**Authors:** Mohamed Boubekri, Jaewook Lee, Piers MacNaughton, May Woo, Lauren Schuyler, Brandon Tinianov, Usha Satish

**Affiliations:** 1 School of Architecture, University of Illinois Urbana-Champaign, Champaign, IL 61820, USA; jlee764@illinois.edu; 2 Department of Environmental Health, Harvard T.H. Chan School of Public Health, Boston, MA 02115, USA; piers.macnaughton@view.com; 3 View Inc., Milpitas, CA 95035, USA; may.woo@view.com (M.W.); Lauren.Schuyler@view.com (L.S.); brandon.tinianov@view.com (B.T.); 4 Psychiatry and Behavioral Sciences, SUNY Upstate Medical University, Syracuse, NY 13210, USA; satishusha27@gmail.com

**Keywords:** daylight, views, health building, cognitive function, productivity, sleep

## Abstract

A growing awareness has recently emerged on the health benefits of exposure to daylight and views. Daylight exposure is linked to circadian rhythm regulation, which can have significant impacts on sleep quality and cognitive function. Views of nature have also been shown to impact emotional affect and performance. This study explores the impact of optimized daylight and views on the sleep and cognitive performance of office workers. Thirty knowledge workers spent one week working in each of two office environments with identical layouts, furnishings, and orientations; however, one was outfitted with electrochromic glass and the other with traditional blinds, producing lighting conditions of 40.6 and 316 equivalent melanopic lux, respectively. Participants in the optimized daylight and views condition slept 37 min longer as measured by wrist-worn actigraphs and scored 42% higher on cognitive simulations designed to test their higher order decision-making performance. Both sleep and cognitive function were impacted after one day in the space, yet the impacts became more significant over the course of the week. The positive effect of optimized daylight and views on cognitive function was comparable for almost all participants, while increases in sleep duration were significantly greater for those with the lowest baseline sleep duration. This study stresses the significance of designing with daylight in order to optimize the sleep quality and performance of office workers.

## 1. Introduction

In the early days of the solar architecture movement, the use of daylighting was advocated primarily based on its potential to reduce building energy consumption. In practice, however, this argument has not experienced the widespread adoption anticipated by the industry; most buildings built in the last 20 years continue to underutilize daylighting solutions and for the most part, architects and developers have favored energy efficient solutions promoted by the electric lighting industry. Although studies have explored the relationship between daylighting and building occupant wellbeing, office worker productivity, and children’s scholastic performance [1,2,3], only a limited number of these studies have focused on quantifying the impact of daylight at the workplace on such specific health indicators as vitamin D deficiency, sleep quality, stress, emotional wellbeing, or cognitive function.

One of the key impacts of light exposure is its effect on the human circadian clock, which influences the body’s sleep-wake cycles. Exposure to daily light and dark patterns is one of the main environmental cues that maintain circadian rhythms [4] as it signals the brain when to secrete hormones such as cortisol and serotonin, which prepare the body for the day’s activities, and when to produce hormones such as melatonin, which trigger sleep onset. This diurnal cycle controlled by the alternance of light and darkness regulates many of our vital bodily functions including our body temperature, hormone secretion, and blood pressure. This cycle is regulated by our exposure to natural light throughout the day. However, in this modern society where we spend 87% of our time indoors [5], indoor light levels should ideally mimic the characteristics of light provided by the natural environment—its intensity, spectral characteristic, timing, and exposure duration—which are needed to maintain circadian rhythms and ensure proper sleep and health. 

Sleep quality and quantity are important health indicators much like our vital signs as they impact our mood, cognitive performance, and a variety of other health outcomes [6,7,8,9]. Deficient sleep has been associated with leading causes of death in the United States, including cardiovascular disease, malignant neoplasms, cerebrovascular disease, work accidents, diabetes, and hypertension, among others [10,11]. Despite the crucial role that sleep plays in our health, a recent study of nearly 400,000 respondents revealed that Americans are sleeping less than they did a decade ago; in 2017, 32.9% of Americans were sleeping six hours or fewer, compared to 28.6% in 2004 [12]. Considering the essential role of daylight on circadian rhythm, there is strong evidence linking daylight to sleep and general health indicators. A comparison of sleep time of two groups of office workers, one having minimal or no exposure to daylight and another with windows and plenty of daylight at their workstations, showed that the group with windows slept on average 46 min longer per night than the group without windows [13]. This same study revealed that workers without windows had significantly worse scores on two dimensions of the Short Form 36 (SF-36) Health Survey—role limitation due to physical problems and vitality compared to those with windows. Another recent study exploring the impact of daylight in elementary school classrooms found that children exposed to plenty of daylight through large windows slept 36 min longer than children exposed to little daylight in their classroom [14]. There is also evidence that links insufficient sleep to a range of significant impairments such as memory loss, slower psychomotor reflexes, and diminished attention [15,16,17], all of which are markers of good or bad performance. Early morning awakening was independently and significantly associated with worse executive functioning [18].

In addition to daylight, views to the outdoors have been shown to offer psychological and health benefits to building occupants. Just as many studies have shown that gardens have a healing power, providing views of green landscapes and vegetation seems to induce similar restorative benefits. This has been demonstrated in a study of patients recovering from surgery in an intensive care unit where patients with access to windows overlooking a green landscape had shorter post-operative stays, had fewer negative evaluations by nurses, and took fewer pain medications than patients whose windows faced a brick wall [19]. Numerous other studies have revealed the psychological benefits of having a view in a work environment; people tend to express more negative feelings about their workplaces, have increased drowsiness and stress, and reduced cognitive function when working in windowless or underground environments [20,21,22,23,24].

The building façade is an intermediary between the indoor and outdoor environments, filtering the amount of daylight that people receive inside buildings. By way of their shapes and fenestration systems, buildings play a significant role in determining the extent of daylight and views that people are exposed to at any given moment. However, designing the façade to strike a balance between daylight penetration, access to views, visual and thermal comfort, and energy use is complex as standard solutions each have their consequences. Moveable window shades allow some level of control of daylight access to promote circadian rhythm entrainment and health, but block occupants from having a view of the outdoors; electric lighting solutions alone cannot provide the necessary light levels for circadian rhythm entrainment without excessive energy consumption. Harnessing and optimizing daylight exposure therefore presents a practical approach for achieving proper circadian rhythm entrainment and by doing so, unlocks significant potential to positively impact occupant health while meeting sustainability goals. 

One of the most promising advances recently made in the area of building enclosure systems is the advent of electrochromic (EC) glazing technology. EC technology enables thin coatings embedded within two or more layers of glass to change from a clear to tinted when a low-voltage electric DC current is applied. As the transparency of the glass is altered, so are the visible and heat transmissivities of the glass, causing a nearly instantaneous dynamic change in the spectrum and the intensity of the light passing through the glass. In addition to the dynamic control of daylight to mitigate glare, EC technology also increases the amount of lighting in the blue range of the visible spectrum that is optimal for circadian rhythm entrainment.

In this study, 30 participants spent 1 week working in each of two identical office suites, with the single distinction being the use of EC glass or traditional blinds as the window shading mechanism. Our overarching hypothesis is that optimized daylight conditions, achieved by use of EC glass, results in improved sleep and improved real-world productivity among office workers. 

## 2. Materials and Methods 

### 2.1. Study Design

**Figure 1:**
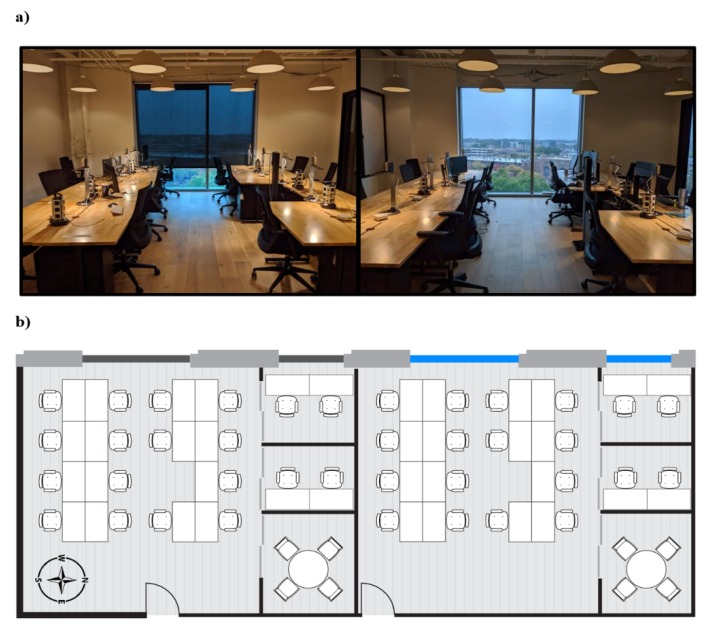
(**a**) Photos and (**b**) floorplans of the two office environments.

This study utilized a case-crossover design to test the effect of exposure to daylight and views during office hours on objectively measured cognitive function and sleep. Participants spent one week in each of two office environments: one with traditional roller blinds and the other with EC glazing that would automatically tint when there was direct solar radiation on the façade (Figure 1a). The order of conditions was randomly assigned.

### 2.2. Study Population

30 knowledge workers were recruited from the Durham, North Carolina area to participate in a multiweek study of their sleep and cognitive function. The study population was restricted to non-sensitive working individuals between the ages of 21 and 65 by excluding people with sleep apnea, insomnia, seasonal or chronic depression, claustrophobia, or schizophrenia. Eligibility was also restricted to those with a general Monday to Friday, eight hours a day work schedule and those who primarily conduct work on a computer. We recruited participants during study information sessions at two coworking offices in the Durham area, including the Durham ID building, which were advertised through flyers and e-mail notifications that were sent to building occupants. Participants were not informed of the experimental conditions or hypotheses in any of the information sessions or study correspondences. The study protocol was reviewed and approved by the University of Illinois Institutional Review Board at Urbana-Champaign. All participants signed informed consent documents and received a nominal stipend for participation in this study. 

The participants relocated to office suites in the Durham ID building for two workweeks in November of 2019. The participants were randomly assigned to one of the two test offices for the first week and then switched to the other office for the following week. The demographics of the two groups were similar in terms of sex, age, education, race, job function, and baseline health (Table 1). They conducted their normal office work from 9:00 am to 5:00 pm with lunch provided within the premises at noon on each day. Each office suite had three breakout rooms (Figure 1b) for participants to conduct phone calls without having to leave the space, as well as printing and bathroom access located on the same floor. 

### 2.3. Environmental Conditions

The two office suites were adjacent to one another and had an identical floor plan and furniture layout. Four rows of workstations were arranged perpendicular to the window. Environmental conditions were assessed using Awair Omni devices which measure temperature (Fahrenheit), relative humidity (%), air quality (CO_2_ in ppm, PM2.5 in µg/m^3^, and TVOCs in µg/m^3^), noise (dB), and light levels (lux) every 5 min. The devices were arranged such that every group of four desks would have two devices on the workstation oriented upwards to measure horizontal task illuminance, with one device at eye level oriented north for vertical luminance of the two participants facing north in that block of four desks and one device at eye level oriented south for vertical luminance of the two participants facing south. In addition, a spectrometer (LI-COR model LI-180) was located centrally in each office, facing westward towards the window. The spectrometer measured lux, correlated color temperature (CCT), color rendering index (CRI), and photon flux density at every nanometer band from 380 nm to 780 nm every 5 min. 

**Figure 2:**
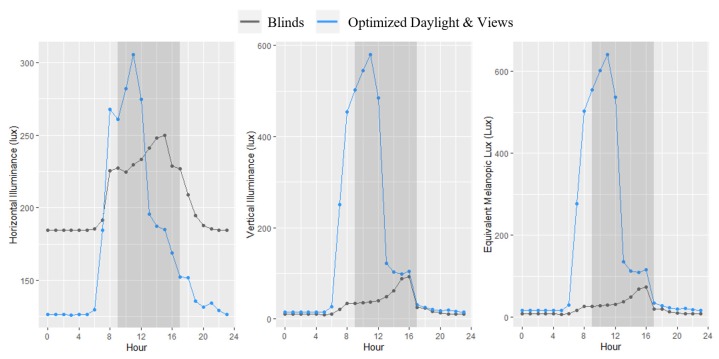
Daily variation in horizontal illuminance, west-facing vertical illuminance, and west-facing vertical equivalent melanopic lux in the two office environments.

The offices had a common HVAC system with individual variable air volume units in each office to maintain temperature set points. Overhead lighting was kept consistent in both offices and the workstations were also outfitted with task lighting set to a consistent brightness and color temperature. The electric lighting was designed to provide an adequate surface illuminance greater than 150 lux in the absence of any daylight. As a result, thermal, air quality, noise, and nighttime horizontal lighting conditions in the two offices were similar across the study period (Figure 2 and Table 2).

The one difference between the two test conditions was the façade configuration (Figure 1a). One office suite had both of its windows outfitted with 1.5% visible transmittance dark fabric roller blinds, which were kept at a constant position of 75% deployed such that they were just below desk level. The positioning assured that there would not be any direct solar glare on the workstations. It also aligns with previous research showing that when blinds are being used, they are deployed 75% of the way down or more in 63% of instances [25]. The occupants were still able to see outside through the blinds as shown in Figure 2. The windows in the other office suite had electrochromic glazing technology. The glass has four tint states, ranging from a visible transmittance of 58% down to 0.5%, becoming increasingly dark and shifting toward shorter wavelengths at higher tint states as evidenced in the afternoon illuminance and CCT levels (Figure 2). However, due to the fact that human vision perceives changes in light logarithmically, even the darkest tint state maintains a clear view to the outside. A predictive algorithm factors in the orientation of the window relative to the sun and local cloud cover via a sensor located on the roof to tint the glass when a glare condition is present. As the office had a west orientation, the tint state of the glass was typically clear (58% light transmittance) from 9:00 am to 12:30 pm, at which time it would tint to its darkest tint state (0.5% light transmittance) over the course of 30 to 60 min. The afternoon tint state was dependent on the level of cloud cover on each day. 

**Figure 3:**
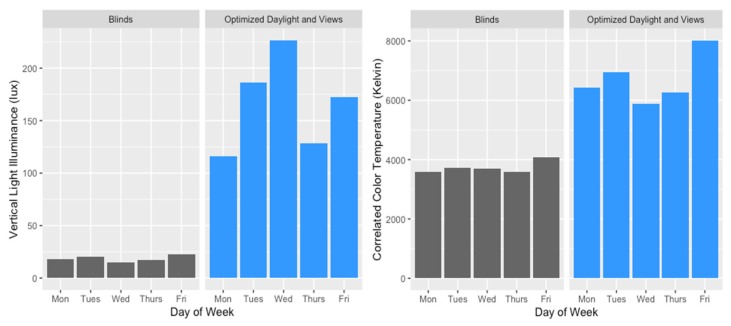
Comparison of daily exposure to vertical light illuminance and correlated color temperature (CCT) across the study conditions.

Light measurements taken at participants’ desks reveal large differences in horizontal and vertical illuminance exposures across the two test conditions (Figure 2 and Figure 3). Moreover, the correlated color temperature (CCT) of the daylight penetrating into the office with EC glass was much higher than in the office with blinds, with levels between 7500 K and 8700 K throughout the morning hours that then dropped below 5000 K after 4:00 PM (Figure 2 and Figure 3). In the blinds office, the CCT remained below 5000 K during working hours and dropped below 3750 K after 4:00 PM. CCT between 7500 K and 8700 K as observed in the office with optimized daylight translates to a wavelength peaking closer to the blue wavelength range (450 nm) that is more ideal for circadian rhythm entrainment during the morning hours. Conditions were relatively constant over the course of the study within each office (Figure 3).

To translate the environmental lighting conditions to physiologically relevant values, we estimated equivalent melanopic lux (EML) from illuminance and CCT as measured by the desk sensors and spectrometers. EML weights the irradiance by the spectral sensitivity of melanopsin, which is a photoreceptor in the human eye and is considered a better predictor of circadian impact than photopic flux [26,27]. EML was estimated using the methodology and irradiance calculator presented by Lucas et al. [28], where the illuminance in each direction in each of the offices over the course of the day was weighted by the spectral power distributions for the following light sources: daylight [D65 – CCT of 6500 K] for the room with optimized daylight and views, and LED [White LED – CCT of 4730 K] for the room with blinds. These light sources were chosen for approximation based on closest CCT value and the nature of the lighting conditions in each room. In addition to EML, Circadian Stimulus (CS) was calculated according to Rea et al., which estimates the effective photic stimulus for the circadian system, varying between a threshold level of 0.1 to a saturation level of 0.7 [29]. An exposure to CS of 0.3 or higher for one hour or more in the early part of the morning has been shown to effectively maintain office workers’ circadian system [30].

### 2.4. Daily Surveys

At the end of every workday and prior to leaving their workstation, participants were asked to complete daily surveys relating to their lifestyle, behaviors, health, metacognitive status, and environmental perceptions. They also completed baseline and weekly surveys about general health factors and experiences in each work environment. These surveys were administered electronically using the Qualtrics platform. 

### 2.5. Sleep Assessment

Participants wore a wrist-worn actigraph device over the course of the study, including weekends. The ActiGraph wgt3x, a research-grade 3-axis accelerometer, was used to collect raw acceleration data at a 30 Hz sampling frequency. ActiGraph’s proprietary software, ActiLife, was then used to post-process the raw acceleration data into activity metrics (including steps, kCals, inclinometer, and active and sedentary bouts) and sleep metrics (including total sleep time, estimated sleep and wake times, sleep efficiency, sleep latency, and wake time after sleep onset). ActiLife’s default Cole-Kripke algorithm was used to score sleep from the activity data given its validity against polysomnography and its relevance to adult populations [31]. The actigraph also contained an ambient light sensor that continuously collected personal light exposure at each participant’s wrist (lux) and a wear sensor for compliance monitoring. Sleep data meeting the following criteria were included in the analysis: sleep periods following the workday when the participant was present and at their assigned workstation for at least 6 h out of the 8 h workday, sleep periods that represented the work weeknights (Monday through Thursday nights), and sleep data from a subset of 24 participants after the exclusion of 6 participants who noted significant young childcare responsibilities; having been prescribed anxiety, ADHD, or sleep medication in the past year; and who had abnormal sleep patterns, characteristics that were deemed potential confounders. 

### 2.6. Cognitive Assessment

At 3:00 PM on the Monday and Friday of each test week, participants completed the Strategic Management Simulation (SMS) assessment, which is a validated, computer-based simulation software [32,33]. Briefly, the simulation would place the participant in a decision-making role, such as an emergency planner or city mayor, and over the course of the 83 min simulation, they would be tasked with responding to events that occur under their purview. The participants only had a short contextual reference document that provided baseline information about the scenario for that day. The scenario was unique for each day of testing, and previous research has shown high repeat validity and no progressive learning effect between scenarios [34]. All actions taken throughout the simulation were processed by the SMS software to provide scores on nine cognitive domains. These domains range from basic (activity levels, information search) to complex (crisis response, information management, and strategy) and span various cognitive styles that collectively integrate into higher order decision-making performance. Unlike other cognitive tests, which isolate individual mechanistic processes (e.g., working memory, attention), the SMS assessment provides a composite approach using real-world scenarios, which ultimately leads to stronger correlations with other indicators of job performance such as salary at age, number of employees supervised, and number of promotions [33]. 

At the start of each assessment, participants were asked to put away their work activities and close out of other programs on their computer. They were provided individual logins to the software to commence the simulation. Each office had a member of the study team supervising for the duration of the assessment to answer questions and ensure participants remained focused on the test. A technical expert on the SMS tool was available during all assessments to assist with the administration. On the first Monday of the study, participants watched an informational video tutorial describing the user interface and experience to provide familiarity with the software.

### 2.7. Statistical Analysis

Generalized additive mixed effect models were used to test associations between lighting conditions, sleep, and cognitive function. Participant ID was treated as a random intercept to control for inter-personal confounders. The final sleep model is shown in Equation (1).

Total Sleep Time = β_0_ + β_1_∙Condition + β_2_∙Melatonin + β_3_∙Exercise + β_4_∙Alcohol + β_5_∙Caffeine + β_6_∙Screen + e_i,j_ + u_i_. (1)

Use of melatonin, exercise engagement during the evening prior to the sleep period, and intake of caffeine after 12:00 PM the day preceding the sleep period were treated as binary variables, and the number of alcoholic drinks and duration of screen time (hours) were treated as continuous variables. These data were self-reported in the daily surveys by participants. The final cognitive model is shown in Equation (2).

SMS Score_norm_ = β_0_ + β_1_∙Condition + s(temp) + e_i,j_ + u_i_. (2)

Cognitive scores were normalized to the Blinds condition to allow for comparisons across cognitive function domains. Temperature was treated as a penalized spline to account for the non-linear relationship between temperature and cognitive scores. Noise, CO_2_, humidity, and particulate matter were not found to be significant variables and were excluded from the model. Analyses were performed using the open-source statistical package R version 3.5.0 (R Project for Statistical Computing, Vienna, Austria).

## 3. Results

### 3.1. Sleep Assessment

Sleep metrics were calculated from the raw acceleration data using ActiGraph’s proprietary software. Among the sleep metrics measured by the Actigraph device, total sleep time—that is, the duration of time asleep, excluding time in bed before falling asleep and time awake during a sleep interruption—has been shown to closely correlate with sleep time measured by polysomnography, the gold standard for sleep measurements [35], and was thus analyzed in the current study. 

**Figure 4:**
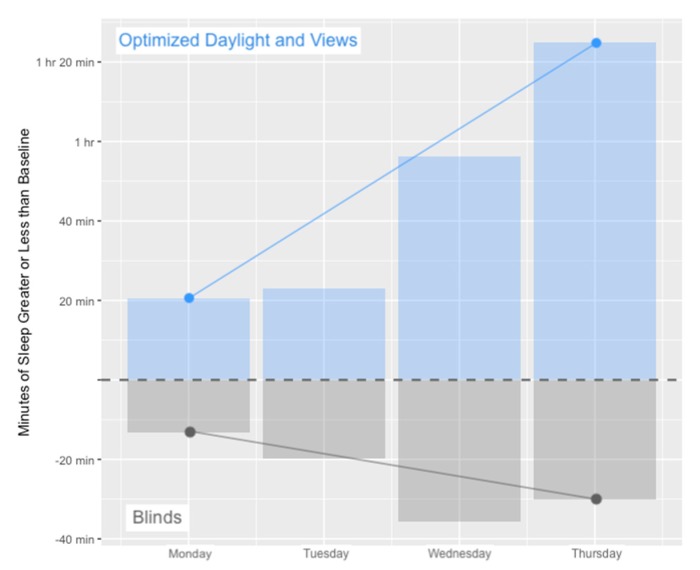
Visualization of the acute (Monday) effect and cumulative (Monday through Thursday) effect of the test conditions on sleep duration, relative to the participant’s measured baseline sleep.

A comparison of sleep time for the 24 participants included in the sleep assessment indicate a significantly longer sleep duration for nights following working in the office with optimized daylight and views. Comparisons within the same day of week across conditions (comparing Monday in the blinds condition to Monday in the optimized daylight & views condition, for example) demonstrate a consistently longer sleep duration in the optimized daylight office, even after only one day of exposure to improved daylighting conditions (Figure 4). 

The mixed effects model controlling for lifestyle factors that are known to affect sleep (melatonin intake, evening exercise, number of alcoholic drinks, caffeine intake after noon, and evening screen time) indicated a statistically significant benefit of 37 min of sleep per night (*p* < 0.001) associated with working in the optimized daylight and views condition (Table 3). In comparison, the effect of melatonin supplement use was associated with an increased sleep duration of 27 min (*p* = 0.396), evening exercise with an increased sleep duration of 22.5 min (*p* = 0.137), alcohol with a loss of 2.5 min per drink (*p* = 0.796), afternoon caffeine intake with a loss of 16 min (*p* = 0.316), and evening screen time with a loss of 7 min per hour of screen time (*p* = 0.313). 

The detrimental effect of moving from the baseline condition and into the office with blinds and the beneficial effect of moving into the office with optimized daylight and views was graphed in order to visualize both the immediate and cumulative effects of the office condition on sleep (Figure 4). The sleep duration greater or less than baseline was calculated for each participant based on the sleep duration measured by their actigraph during the baseline weeks (average baseline sleep across the 24 participants was 6 h 11 min). The immediate effect—that is, the effect of working in the condition for one day—is demonstrated by the shorter sleep duration observed on Monday night in the blinds condition compared to baseline and the longer sleep duration observed on Monday night in the optimized daylight and views condition compared to baseline. The figure also demonstrates the sleep debt or sleep benefit accumulated over the course of the work week in each condition (Figure 4). 

The longer sleep duration in the optimized daylight and view condition was observed in both groups—both the group that was assigned to the blinds condition first and the group that was assigned to the optimized daylight and views condition first. Among those who were assigned to the blinds condition first, participants lost an average 14 min of sleep per night after moving to the blinds office from their baseline condition, then gained an average of 19.7 min after moving to the optimized daylight and views condition. Among those who were assigned to the optimized daylight and views condition first, participants experienced an immediate gain of 21.4 min per night from their baseline condition, then experienced an average loss of 37.7 min of nightly sleep after moving to the blinds office. 

To assess whether the difference in the magnitude of effect of the optimized daylight and views condition across the two groups was due to the group’s order of assigned conditions or characteristics of the group itself, an analysis stratifying by participant’s baseline sleep quality was performed. Sleep quality as measured using actigraphy and confirmed by the PSQI survey upon enrollment indicated a difference in baseline sleep quality across the two groups, although non-significant; the group assigned to the blinds condition first reported slightly better baseline sleep than those assigned to the optimized daylight and views condition first (PSQI global score of 5.46 versus 7.60, see Table 1). Due to this potential incomplete randomization, a stratified analysis based on baseline sleep quality was performed. Nine participants were “good” sleepers and 15 participants were “poor” sleepers, where “good” was classified by a PSQI global score of equal to or less than 5 and “poor” was classified by a PSQI global score of greater than 5. Poor sleepers had a significantly shorter sleep duration in their baseline week, as was expected, but also experienced a significantly greater benefit to their sleep duration when they worked in the office with optimized daylight and views (Table 4).

The mixed effects model stratified by baseline sleep quality indicated that the effect of being in an optimized daylight and views condition was greater in magnitude amongst poor sleepers. Controlling for lifestyle factors, poor sleepers, who started at a shorter baseline sleep duration, gained 52.8 min of sleep (*p* < 0.001), while good sleepers, who started at a longer baseline sleep, gained 18.1 min of sleep (*p* = 0.214) (Table 4).

### 3.2. Cognitive Function

**Figure 5:**
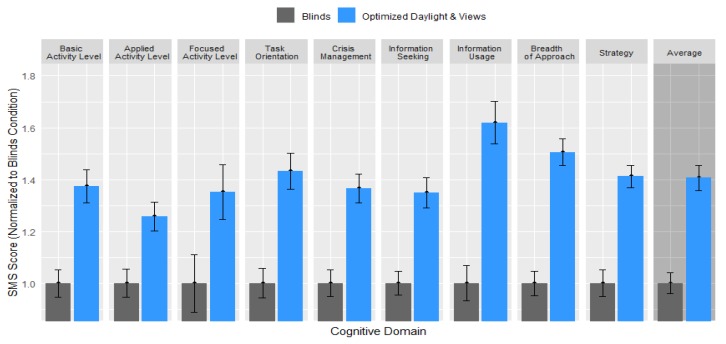
Cognitive scores on the Strategic Management Simulation (SMS) assessment for each of the nine domains of cognitive function, normalized to the Blinds condition.

The results of the SMS tool indicated 26–62% higher cognitive function scores across all nine domains of strategic thinking (Figure 5). These domains, each relating to the ability to set, develop, and fulfill goals and tasks; gather and manage available resources to improve organizational effectiveness; plan actions and strategize successful outcomes; and weather any crisis and emerge with lessons for future success, are critical indicators of work-related performance. 

**Figure 6:**
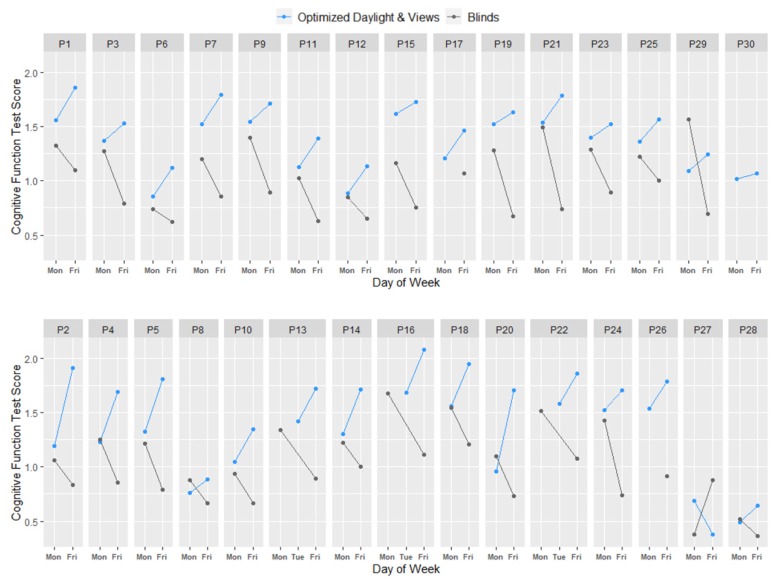
Average cognitive scores on the SMS assessment on each test day, normalized to the Blinds condition, for participants in Group A (top) and Group B (bottom).

Plotting the normalized scores across all four occasions (the assessment taken on Monday and Friday in each test condition) for all 30 participants reveals a high degree of consistency in the effect of the office condition on cognitive function across individuals. The plots suggest both an acute and cumulative benefit of working in the optimized daylight and views condition. Nearly all participants scored higher on the Monday while in the optimized daylight and views condition compared to on Monday while in the blinds condition, demonstrating the acute benefit of optimized daylight and views. Further, all but one participant’s performance improved as they spent the week in the optimized daylight and views condition, while it declined as they spent more time in the blinds condition, demonstrating the cumulative benefit of optimized daylight (Figure 6).

The generalized additive mixed effect model of the effect of the office condition on the normalized cognitive function score, controlling for the effect of the indoor temperature on the day of the assessment, demonstrated that participants scored 42% higher when in the optimized daylight and views condition compared to when they performed the simulation in the blinds condition (*p* < 0.0001) (Table 5).

**Figure 7:**
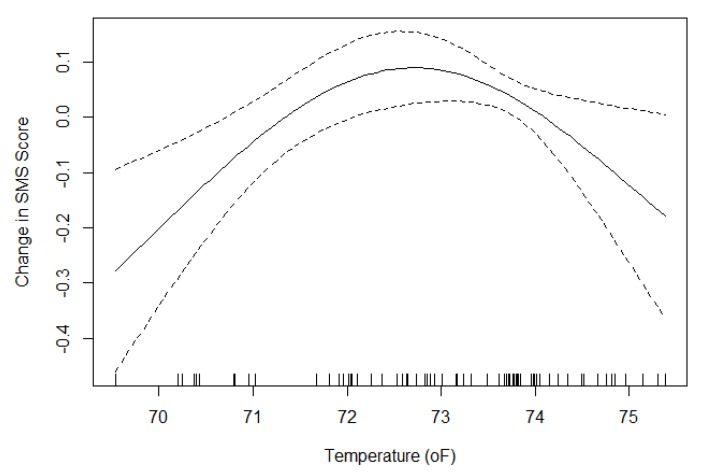
Penalized spline of temperature and average cognitive function scores, controlling for condition and treating participant as a random effect.

Temperature exhibited a non-linear relationship and was kept in the model as a penalized spline (Figure 7). The spline shows a U-shaped curve with optimal cognitive function scores between 72°F and 73°F. In an analysis of the variance of the model, 37.4% of the variability in scores was explained by the random effect for participant, 25.3% was explained by the fixed effects for condition and temperature, and 37.3% was unexplained by the model. 

## 4. Discussion

Participants slept 37 min longer when exposed to optimized daylight and views during the day compared to when they were in an office with traditional blinds, controlling for lifestyle factors. The effect of light exposures was larger than other well-studied factors that impact sleep quality, such as melatonin use, exercise, alcohol consumption, and evening screen use. Both mechanistic models and previous field studies indicate that exposure to short wavelength light, particularly in the morning, is critical for resetting the circadian clock [36,37,38]. We found that providing these lighting conditions in an office setting helped entrain circadian rhythms and increase sleep duration. Surprisingly, the increase in sleep duration occurred even after only one day in the space and then persisted for subsequent days. As a result, participants’ sleep debt accumulated when in the blinds condition compared to their baseline sleep level, while it decreased in the optimized daylight and views condition. The benefit of improved lighting conditions was predominantly experienced by the participants with the worst baseline sleep. The increase in sleep duration was not causing those with adequate sleep to oversleep, but rather ensured that all participants approached a sufficient amount of sleep.

Participants, on average, scored 42% higher (*p*-value < 0.0001) on cognitive assessments in the optimized daylight and views condition compared to the blinds condition. Similar to sleep, cognitive performance demonstrated an acute benefit after one day in the space that compounded with increased time in the space. The effect after one day of optimized daylight exposure was 7%, which could be attributed to the impact of improved views outdoors and to the alerting effects of short wavelength light [39,40]. By Friday, the difference in cognitive function scores was 79%, suggesting a cumulative effect on cognitive performance from daylight deprivation. The improvement in scores between each condition and within each condition from Monday to Friday was highly consistent across all participants, regardless of their baseline performance on the assessment, job function, demographics, or distance from the window. 

The SMS simulation is designed to test real-world productivity rather than performance on specific functional domains of cognitive function like other standardized cognitive batteries. Employees spend the vast majority of their time interacting with organizational stakeholders and completing complex tasks. These require the capacity to design goals efficiently towards optimal goal attainment, to manage the volume of information available with discretion, to use available information towards strategic goals for the organization, and to engage in multidimensional thinking across competing interests. These are evaluated with the SMS along the following clusters of metacognition:

- Task management: the ability to respond to plans and organizational initiatives with optimal goal setting, goal development, and goal fulfillment.
- Information management: the ability to search for optimal information in an efficacious manner and use it appropriately based on situational demands.
- Strategy management: the ability to conceptualize multiple approaches and form systematic plans and actions that are optimally sequenced and integrated toward goal fulfillment.
- Crisis management: the ability to build contingency plans that will enable the organization to cope effectively under exigent conditions.

All these clusters were sensitive to improved daylight and views with particularly pronounced impacts in the information management and strategic domains. 

The study, due to its design to isolate the effects of light levels, was limited in its ability to investigate the effect of other environmental and behavioral variables such as CO_2_, VOCs, ventilation rates, excessive internet use, and recreational drug use that have been shown to influence cognitive performance or sleep [41,42,43,44,45]. However, there was enough variability in temperature between days of the study to see a statistically significant U-shaped relationship between temperature and cognition, consistent with previous research on the topic [46,47]. In addition, we are unable to determine what differences between the two conditions caused the changes in performance. Previous studies indicate that both daylight and views can have positive cognitive benefits, but they are usually perfectly correlated [2]. Because we only tested two conditions, we cannot determine a dose-response relationship between increasing daylight levels or view quality and cognition.

The highly controlled nature of the study design conferred other strengths. In addition to the environmental factors, other potential confounders such as space layout; ergonomic factors; furnishings; and window sizes, placements, and orientations were consistent between the two offices. We provided lunch to keep daytime diet consistent among participants and maintained consistent arrival and departure times in the mornings and evenings. As the offices were collocated, commutes were the same in both conditions. The case-crossover design ensured that the findings were not a result of temporal factors or learning effects. Each person served as their own control to account for baseline differences in sleep quality and cognitive function, giving statistical power to the study despite the small sample size. While participants were not made aware of the purpose of the study, it was impossible to fully blind them to conditions as they were visually different. To avoid potential bias, we used objective assessment methods; sleep duration was measured by actigraphy and cognitive function through a simulation-based assessment. Some of the effect on these outcomes could result from a placebo effect, which nonetheless would be present wherever blinds or EC glass is used in practice.

These design choices improve the internal validity of the study but potentially limit the generalizability of findings. Different office configurations and window treatments will lead to different daylighting conditions. The offices tested in this study compare typical blinds to EC glass, so the impact of windowless offices or offices with no blinds cannot be determined with these data. Previous research has compared windowless rooms to rooms with windows and found similar effects [2,13]. Due to the potential for glare, it is rare in practice to find offices without some type of shading element or window treatment. The orientation of the windows will also impact blind usage and the timing of EC glass tinting. On the east façade, blinds are more likely to be drawn to avoid glare in the morning when people arrive at the office and EC glass will typically be tinted in the morning and clear in the afternoon. Moreover, in our study, we kept the blinds position fixed, which may or may not be the case in other settings. Lastly, we recruited knowledge workers, so these findings cannot be extrapolated to other settings such as schools or hospitals. Previous research has shown similar impacts in those environments.

## 5. Conclusions

This study found that office workers in an office using EC glass to optimize daylight and views slept longer than those working in an office with blinds. In addition, participants also scored 42% higher on cognitive assessments when exposed to optimized daylight and views. The benefits to sleep and cognitive performance were immediate, substantial, and sustained. Since blinds are the predominant glare solution in the market today, this research suggests that there is a significant opportunity to improve sleep quality and office worker performance through the use of EC technology, which optimizes daylight and views while mitigating glare. Developers and architects should consider the substantial benefits of access to daylight and views when designing, constructing, and renovating office buildings.

## Figures and Tables

**Table 1:** Summary of Participant Demographics.

		Total or Average	Group A: Blinds First	Group B: Optimized Daylight & Views First
Sex	Male	19	9	10
	Female	11	6	5
Age	Age (years)	34	36.5	31.5
Race	White/Caucasian	16	8	8
	Black/African American	7	4	3
	Hispanic/Latino in Origin	1	0	1
	Asian	2	1	1
	Multiracial	4	2	2
Education	High school graduate	1	0	1
	Some college	2	1	1
	College degree or higher	27	14	13
Job category	Managerial	4	2	2
	Professional	15	10	5
	Technical	8	2	6
	Secretarial or Clerical	1	0	1
	Other	2	1	1
Lifestyle factors	Childcare duties	8	4	4
	Major housekeeping	16	8	8
	Care for elderly or disabled	1	1	0
	Other (education, etc.)	11	7	4
Medications	Allergy	6	3	3
	Anxiety	4	2	2
	Melatonin	3	2	1
	Pain	3	1	2
	Sleep	2	1	1
Baseline office condition	Daylight rating at work [1,2,3,4,5,6,7,8,9,10]	7.53	7.80	7.26
	Job satisfaction [1,2,3,4,5]	4.13	4.13	4.13
Baseline Sleep (PSQI)	Global Score	6.53	5.46	7.60

**Table 2:** Environmental conditions in the two office environments.

	Blinds	Optimized Daylight & Views
Lighting Conditions		
Horizontal Illuminance (Lux)	234	223
Horizontal EML (Lux)	185	246
Vertical Illuminance—North (Lux)	18.3	143
Vertical EML—North (Lux)	14.5	158
Vertical Illuminance—South (Lux)	26.2	137
Vertical EML—South (Lux)	20.7	151
Vertical Illuminance—West (Lux)	51.4	286
Vertical EML—West (Lux)	40.6	316
CCT (K)	4122	7485
Circadian Stimulus (CS)	0.05	0.42
Indoor Environmental Quality		
Temperature (°F)	72.3	74.1
Relative Humidity (%)	41.4	38.7
CO_2_ (ppm)	998	893
PM_2.5_ (µg/m^3^)	0.76	1.2
TVOC (µg/m^3^)	139	122
Noise (dB)	59.8	58.0

**Table 3:** Linear mixed-effects model of sleep duration by office condition and lifestyle factors (use of melatonin, engagement in exercise, number of alcoholic drinks, caffeine intake after noon, and evening screen time duration) (N_subjects_ = 24, N_observations_ = 147).

Variable	Estimate (Minutes)	*p*-Value
Baseline Sleep Duration (β_0_)	362.21	<0.001
Condition: Optimized Daylight & Views (β_1_)	36.96	<0.001
Melatonin, Yes/No (β_2_)	27.09	0.396
Evening exercise, Yes/No (β_3_)	22.51	0.137
Alcoholic drinks (β_4_)	−2.48	0.796
Caffeine after 12 pm, Yes/No (β_5_)	−16.23	0.316
Evening screen time, hours (β_6_)	−6.85	0.313

**Table 4:** Linear mixed effects model of sleep duration by office condition and lifestyle factors (use of melatonin, engagement in exercise, number of alcoholic drinks, caffeine intake after noon, and evening screen time duration), stratified by baseline sleep quality.

	Good Sleepers		Poor Sleepers	
Variable	Estimate (Minutes)	*p*-Value	Estimate (Minutes)	*p*-Value
Baseline Sleep Duration (β_0_)	390.46	<0.001	344.55	<0.001
Condition: Optimized Daylight and Views (β_1_)	18.08	0.214	52.80	<0.001
Melatonin, Yes/No (β_2_)			18.30	0.599
Evening exercise, Yes/No (β_3_)	−3.87	0.813	25.86	0.297
Alcoholic drinks (β_4_)	19.57	0.062	−22.95	0.132
Caffeine after 12 pm, Yes/No (β_5_)	−39.59	0.020	−10.52	0.648
Evening screen time, hours (β_6_)	−4.71	0.651	−6.19	0.487

**Table 5:** Generalized additive mixed effect model of the effect of condition on average cognitive function scores, normalized to the Blinds condition, controlling for temperature and treating participant as a random effect.

Variable	Estimate	*p*-Value
Intercept (β_0_)	0.99	<0.0001
Condition: Optimized Daylight & Views (β_1_)	0.42	<0.0001
s(Temp)	-	0.006
